# Beyond epithelial damage: vascular and endothelial contributions to idiopathic pulmonary fibrosis

**DOI:** 10.1172/JCI172058

**Published:** 2023-09-15

**Authors:** James May, Jane A. Mitchell, R. Gisli Jenkins

**Affiliations:** National Heart and Lung Institute, Imperial College London, London, United Kingdom.

## Abstract

Idiopathic pulmonary fibrosis (IPF) is a progressive scarring disease of the lung with poor survival. The incidence and mortality of IPF are rising, but treatment remains limited. Currently, two drugs can slow the scarring process but often at the expense of intolerable side effects, and without substantially changing overall survival. A better understanding of mechanisms underlying IPF is likely to lead to improved therapies. The current paradigm proposes that repetitive alveolar epithelial injury from noxious stimuli in a genetically primed individual is followed by abnormal wound healing, including aberrant activity of extracellular matrix–secreting cells, with resultant tissue fibrosis and parenchymal damage. However, this may underplay the importance of the vascular contribution to fibrogenesis. The lungs receive 100% of the cardiac output, and vascular abnormalities in IPF include (a) heterogeneous vessel formation throughout fibrotic lung, including the development of abnormal dilated vessels and anastomoses; (b) abnormal spatially distributed populations of endothelial cells (ECs); (c) dysregulation of endothelial protective pathways such as prostacyclin signaling; and (d) an increased frequency of common vascular and metabolic comorbidities. Here, we propose that vascular and EC abnormalities are both causal and consequential in the pathobiology of IPF and that fuller evaluation of dysregulated pathways may lead to effective therapies and a cure for this devastating disease.

## Introduction

Idiopathic pulmonary fibrosis (IPF) (1) is a progressive fibrotic lung disease with a poor prognosis (2). As fibrosis progresses there is worsening gas exchange with consequent increasing dyspnea, development of respiratory failure, and ultimately death. The adjusted incidence and prevalence of IPF globally are estimated to be between 0.09 and 1.30 and between 0.33 and 4.51 per 10,000 persons, respectively (3), and the incidence is rising (4). The poor prognosis of IPF equates to a median survival of just 3 to 4 years, although survival may improve with antifibrotic therapies (5, 6). At present, there are only two licensed therapies, pirfenidone and nintedanib, that slow the rate of decline in forced vital capacity (FVC) and increase progression-free survival (7, 8).

The pathogenesis of IPF is considered to result from injury to the alveolar epithelium by a range of insults including cigarette smoke, gastric acid, air pollution, and viruses in a genetically primed individual, driving aberrant activity of mesenchymal cell populations (9). Subsequent production of extracellular matrix (ECM) components including collagens and fibronectin results in interstitial fibrosis, alveolar collapse, and loss of effective lung tissue (10). Many of the genes that confer increased risk of disease development are associated with alveolar epithelial cells (11). Similarly, telomere shortening in alveolar epithelial cells is sufficient to promote the development of pulmonary fibrosis in mice (12). Furthermore, biomarkers that reflect epithelial injury are associated with poorer outcomes in patients with IPF (13). Because of the intimate anatomical proximity of the alveolar epithelium and endothelium, injury to the alveoli will also result in vascular damage. Indeed, Margaret Turner-Warwick highlighted the importance of the vasculature over 50 years ago and first described the development of digital clubbing, an important vascular phenomenon associated with IPF (14, 15). Furthermore, one of the major targets of nintedanib is the receptor for VEGF, a potently angiogenic molecule. Consistently, recent trials have identified potential antifibrotic effects of treprostinil, which mimics the cardioprotective hormone prostacyclin, in IPF (16). Finally, the importance of vascular comorbidities and their treatment in pulmonary fibrosis is emerging (17–19). It is, therefore, likely that the pulmonary vasculature may have a prominent role in the pathogenesis of IPF and the systemic vasculature in cardiovascular comorbidities.

## The vasculature in IPF

**Figure 1:**
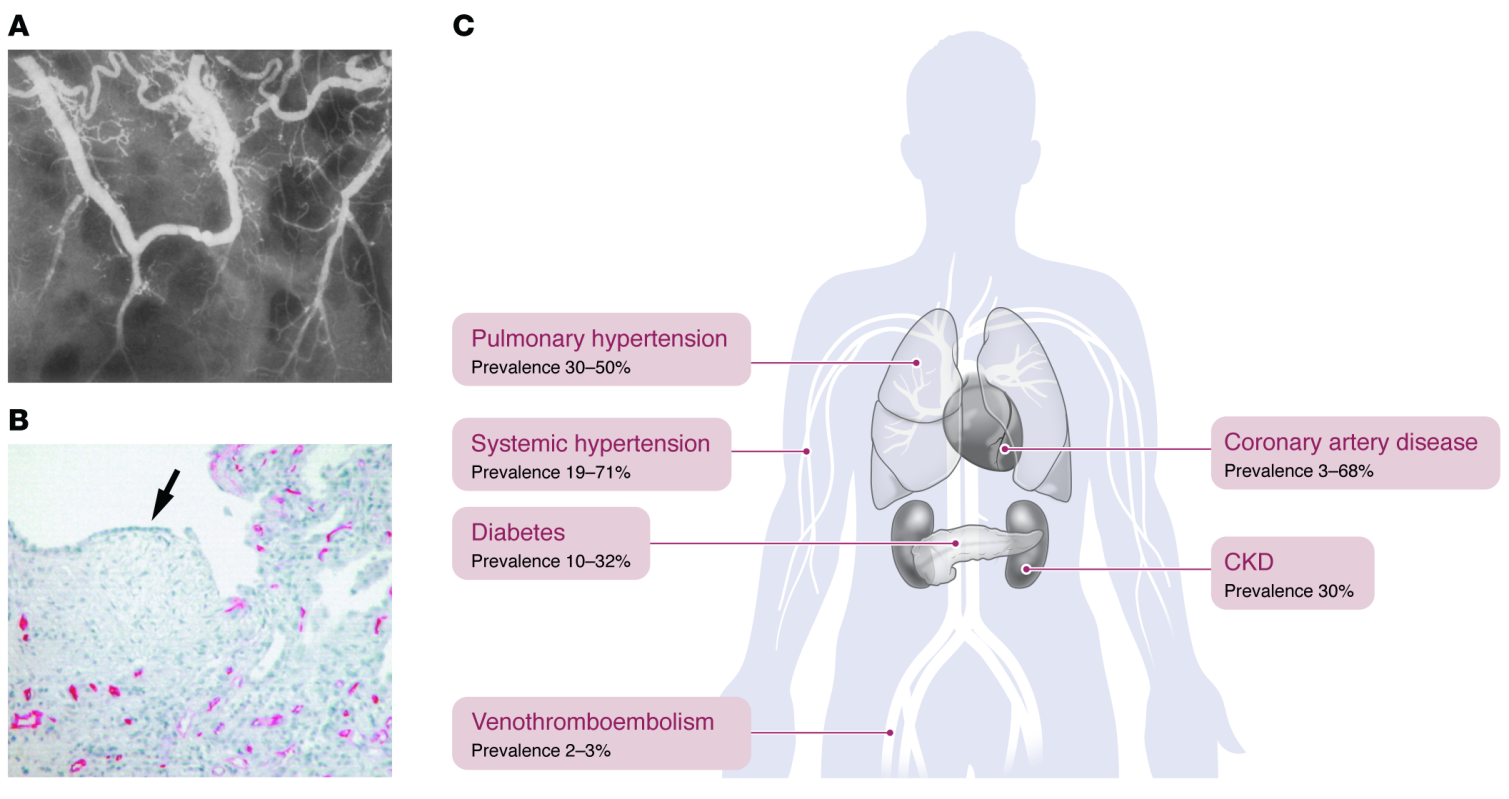
Vascular abnormalities in IPF are reflected within cells, tissue, and the entire organism. (**A**) Pulmonary-bronchial anastomoses often develop in IPF and can be visualized by radiography. In healthy tissue, vessels communicate through the capillary bed, and there is an absence of these larger, tortuous communications (23). (**B**) Fibroblastic foci from fibrotic parenchyma lack ECs (as indicated by an absence of staining for CD34), confirming a lack of vascularity. In healthy tissue, ECs line the vessel walls and are distributed throughout lung tissue. Reproduced with permission from the American Thoracic Society (159). (**C**) Vascular comorbidities associate with IPF, suggesting that ECs contribute to and are affected by fibrosis.

Vascular abnormalities in IPF include the development of vascular connections known as pulmonary-bronchial artery anastomoses and are accompanied by abnormal populations of endothelial cells (ECs) and vascular comorbidities such as pulmonary hypertension (PH) and coronary artery disease (20) (Figure 1). However, targeting vascular-relevant pathways has yielded conflicting results (1, 21, 22) (Table 1). Whether vascular abnormalities promote IPF or are a consequence of progressive fibrosis remains controversial. This Review summarizes the current understanding of the vasculature in IPF and provides support for the concept that vascular injury promotes fibrosis.

## Cellular mechanisms

### Vascular remodeling.

Margaret Turner-Warwick described the expansion of the vasculature with numerous pulmonary-bronchial arterial anastomoses in diffuse pulmonary fibrosis as early as 1963 (23) (Figure 1). These abnormally dilated large vessels are found in the periphery of honeycomb cysts. However, there is marked heterogeneity in fibrotic tissue, and, in particular, areas of minimal fibrosis have been shown to have dense capillary networks whereas the most scarred tissue, rich in fibroblastic foci, almost complete lacks vasculature (Figure 1) (24). These vasculature changes correspond with a relevant growth factor gradient, with VEGF having almost undetectable levels in the regions of worst scarring (25) and showing higher concentrations in areas of relatively preserved lung tissue (26). In some animal models, inhibition of VEGF protects against bleomycin-induced lung injury (27). Conversely, and somewhat contradictorily, VEGF overexpression in transgenic mice attenuated the fibrotic effect of bleomycin, and in vitro application of VEGF to apoptotic ECs prevented an injurious signal to epithelial cells (28).

**Figure 2:**
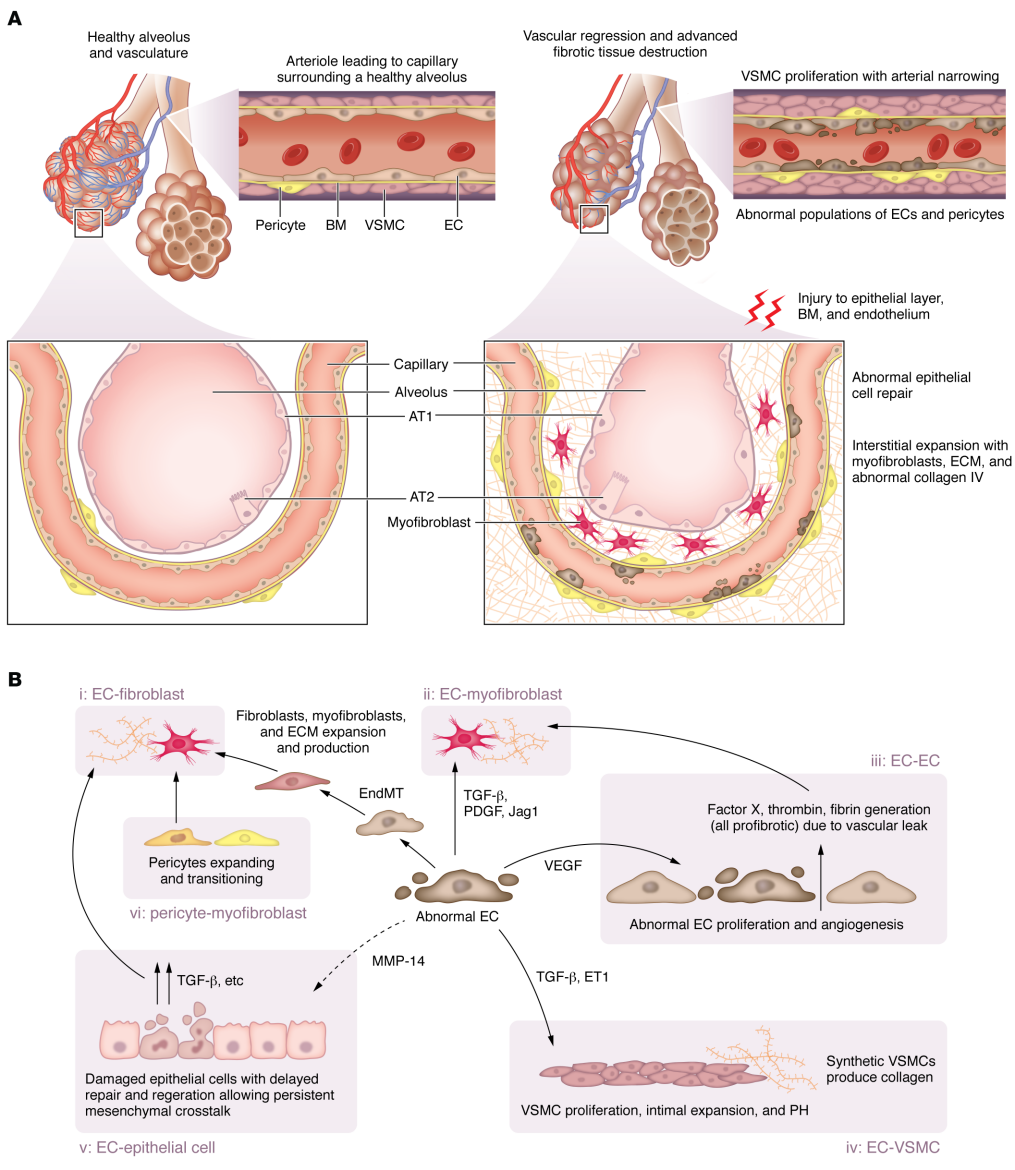
ECs support healthy vasculature and undergo dramatic changes in IPF. (**A**) Damaged epithelium releases active TGF-β and other profibrotic mediators. The original injury also disrupts the BM and the neighboring endothelial layer, which responds to the profibrotic signal. ECs subsequently secrete similar profibrotic mediators and lose the ability to synthesize protective hormones such as eNOS and prostacyclin. This process can stimulate VEGF production, which drives EC proliferation, and ECs distributed throughout the lung propagate fibrosis. Compared with healthy lungs, IPF lungs have a higher proportion of apoptotic ECs, fibroblasts, pericytes, and VSMCs. Cellular proliferation and newly generated vessels expand affected lung tissue. With progressive vascular pathology there is ultimately advanced tissue destruction, and eventually vascular regression develops in the fibroblastic foci. (**B**) In IPF, the EC participates in several cell-cell interactions and cell transitions. Damaged ECs produce factors that signal to other ECs and promote damage or drive the transition to other cell types: (i) EC-fibroblast: ECs transition into a fibroblast-type cell via EndMT to contribute to the pool of profibrotic cells. (ii) EC-myofibroblast: Damaged ECs also secrete TGF-β, PDGF, and Jag1 to enhance fibroblast-myofibroblast transition and ECM secretion. (iii) EC-EC: Abnormal ECs secrete VEGF, which promotes EC proliferation and abnormal vessel formation, thus contributing to the pool of ECs that can propagate this process. Compromised tight junctions leak coagulation factors, driving fibrosis. (iv) EC-VSMC: EC production of TGF-β and ET1 promotes VSMC proliferation, contributing to PH and a switch to a synthetic phenotype. (v) EC–epithelial cell: Downregulation of protective factors such as MMP-14 delays epithelial repair, allowing persistent epithelial-mesenchymal crosstalk. (vi) Pericyte-myofibroblast: Disrupted Wnt signaling associated with ECs drives pericytes to transition into a myofibroblast-type cell.

It is, therefore, unclear whether the VEGF-inhibiting effects of nintedanib are undermining or augmenting its antifibrotic effects. In advanced disease states, the overall effect is a net reduction in pulmonary vascular surface area, which, in part, explains the increased frequency of PH. This loss of pulmonary vascular surface area is associated with distinct cellular changes in ECs, vascular smooth muscle cells (VSMCs), and pericytes, all of which may directly contribute to the pathogenesis of IPF (Figure 2).

### Endothelial cells.

Alveolar injury is likely to directly injure ECs (Figure 2A). ECs share a basement membrane with the alveolar epithelial cells and are key components of the alveolus, comprising approximately 30% of pulmonary cells in normal lung (29). Bleomycin studies modeling lung injury in mice have demonstrated that ECs upregulate profibrotic molecules such as plasminogen activator inhibitor-1 (PAI-1), TGF-β, and PDGF and lose their ability to generate nitric oxide synthase (NOS) and prostacyclin (30). In IPF, there is evidence that epithelial cell injury results in release of active TGF-β, which can activate ECs, causing an imbalance of angiostatic and angiogenic mediators including VEGF, resulting in abnormal EC proliferation and apoptosis (31) (Figure 2).

In IPF, an abnormal, ectopic population of COL151A-expressing ECs have been identified using single-cell RNA sequencing (32). These COL151A cells are normally confined to the peribronchial and subpleural regions of large airways but are never embedded within parenchyma in healthy states. However, they are found in larger numbers in more distal lung tissue and especially areas of fibrosis in patients with IPF. Antibodies targeting ECs that can induce microvascular injury and accelerate EC necrosis have also been identified in the sera of patients with IPF (33). Similarly, circulating ECs, serving as markers of EC damage, are found in higher concentrations in the sera of patients with IPF, while myeloid progenitor cells are found at lower concentration (34).

Numerous animal models have shed light on the role of the endothelium in pulmonary fibrosis. In one mouse model, pulmonary capillary ECs have been shown to support epithelial cell proliferation and alveolar regeneration through the secretion of factors including MMP-14, a process that is dependent on the activation of VEGF receptors present on ECs (35). Bleomycin-induced lung injury downregulates protective EC signals and recruits profibrotic macrophages that signal through ECs via the Wnt/β-catenin pathway to secrete profibrotic factors including Jag1 (36). These factors activate Notch signaling in neighboring fibroblasts and drive fibrogenesis (36). In similar models, EC autocrine signaling, through sphingosine-1-phosphate (SIP1) GPCRs, is critical in maintaining endothelial barrier integrity and tight junction formation. Disrupted barrier integrity results in increased vascular permeability, exuberant coagulation, and worsened fibrosis following bleomycin administration (37). The endothelial transcription factor ETS-related gene (ERG) is implicated in the generation of regenerative capillary ECs following injury. In bleomycin models, there is evidence that the dysregulated ERG pathway in aging cells is associated with accelerated fibrosis (38). Abnormal ERG signaling is additionally associated with endothelial paracrine signaling through the secretion of connective tissue growth factor (CTGF) that enhances the activity of neighboring fibroblasts that drive tissue remodeling.

ECs may also be directly involved in fibrogenesis through endothelial-mesenchymal transition (EndMT) whereby they develop a contractile, α-smooth muscle actin–expressing phenotype (39). This effect can be induced by the profibrotic mediator TGF-β and is exaggerated when the cells are exposed to hypoxic conditions with consequent induction of HIF-2α (40). EndMT has been further evaluated in mouse models of fibrosis. In a transgenic model that tracked cells following bleomycin injury, accumulated cells identified as having endothelial origin contributed to nearly 20% of the total cell population in areas of active fibrosis (41). A proportion of these cells, 16%, coexpressed collagen I, suggesting they were in a state of transition to a myofibroblast-like cell. Another study has shown that EndMT is dependent on the binding of sterol regulatory element–binding protein 2 (SREBP2), a key protein in cholesterol homeostasis, to specific promoter regions (42). Notably, SREBP2 is upregulated in ECs from bleomycin and IPF models.

These data suggest that following lung injury, damaged ECs secrete profibrotic mediators and are capable of migrating to the parenchymal regions of lung and transdifferentiating into pathogenic myofibroblasts. This model implies that ECs play a fundamental role in pathogenesis, serving as much more than mere bystander cells.

### Vascular smooth muscle cells.

VSMCs are highly contractile cells that constitute the structural form of blood vessels. There is abnormal proliferation and distribution of smooth muscle cells in IPF tissue (43), and under the influence of the tissue microenvironment and growth factors including PDGF and TGF-β, VSMCs may develop a synthetic phenotype capable of producing multiple components of the blood vessel wall, including collagen (44). In vitro studies show upregulation of markers representing a switch from contractile to synthetic phenotype in response to TGF-β stimulation (45). In addition, TGF-β from damaged epithelium and PDGF released from apoptotic ECs stimulate the proliferation of VSMCs (as well as fibroblasts). The cell expansion thickens the intimal layer of the pulmonary artery and arterioles, generating resistance and subsequent PH. VSMC proliferation is additionally driven by other mediators, including CTGF as demonstrated in bleomycin models (46).

VSMCs isolated from patients with IPF show a hyperproliferative state and produce more collagen I via induction of reactive oxygen species compared with cells from control donors. This effect can be blunted by the antifibrotic therapeutic pirfenidone (47). The proliferative, contractile, and synthetic properties of VSMCs are dysregulated in IPF and, therefore, have the potential to contribute to parenchymal fibrosis as well as associated PH.

Adenoviral overexpression of TGF-β1 in the lungs of mice leads to signaling crosstalk between ECs, VSMCs, and fibroblasts, which not only results in increased rates of apoptosis of ECs but also induces activation and proliferation of VSMCs. Importantly, defective bone morphogenetic protein receptor 2 (BMPR2) signaling has been implicated in VSMC activation and proliferation (48). In particular, restoration of the BMPR2 pathway attenuates fibrosis and reduces VSMC activation and proliferation. Further, genetic defects in BMPR2 are well recognized in pulmonary arterial hypertension (PAH) and are present in IPF, supporting a link between the two disease processes.

### Pericytes.

Pericytes are mesenchymal cells, closely related to fibroblasts, that form extensive physical contacts with ECs (e.g., within capillaries) with which they share a basement membrane (49). Pericytes are activated by cyclical mechanical stretch and are fundamental to normal alveolar development (50). Lineage tracing in bleomycin lung injury models demonstrated that this cell population may contribute substantially to the myofibroblast pool in pulmonary fibrosis (51). In particular, there is evidence that signaling disruption in the Wnt pathway occurs simultaneously in ECs and pericytes (52). In IPF, the changes in pulmonary mechanobiology and the phenotypic shift of ECs and VSMCs may have a profound profibrotic effect on pericytes, leading to a rapid expansion of the potently fibrogenic myofibroblasts.

### The alveolar capillary basement membrane.

The alveolar epithelial-capillary basement membrane (BM) is a vital scaffold structure that supports normal repair of the alveolar parenchyma, and loss of BM integrity has been known for many years to be a fundamental pathogenic change in IPF (53). Loss of the BM permits direct alveolar epithelial-mesenchymal cell interaction and promotes TGF-β activation and myofibroblast activation (54). Collagen IV is a major component of the BM, and is the main collagen synthesized by ECs, promoting cell adhesion and migration, and serving as a cofactor in NO-dependent angiogenesis (55). Collagen IV is also synthesized by alveolar type 1 (AT1) epithelial cells, which are lost in IPF. Thus, the primary source of collagen IV in IPF are ECs and pericytes, which are positioned to profoundly affect the anatomical location and function of the BM.

IPF fibroblasts can also synthesize collagen IV; and human fibroblasts exposed to TGF-β stimulation deposit abnormal α_1_ and α_2_ collagen IV chains. This change in BM composition limits myofibroblast migration and promotes their survival, which could contribute to myofibroblast persistence and sustained activity in fibroblastic foci (56–58). The persisting cells provide an additional source of abnormal collagen in patients with IPF. Abnormal collagen IV is associated with aberrant angiogenesis, likely due to disrupted EC binding via a compromised integrin–collagen IV interaction (55). The collagen IV–integrin interaction may, therefore, be a key process in the endothelial contribution to IPF. Indeed, the stiff matrix associated with IPF has been shown to lead to integrin-mediated mechanosensing that mediates MMP-2–dependent degradation of collagen IV, which subsequently promotes myofibroblast invasion of the alveolus (59).

It is clear that key components of the pulmonary vasculature become fundamentally altered during fibrogenesis and are likely to contribute to the ongoing progression of fibrosis in IPF. Therefore, understanding the molecular mechanisms that contribute to these cellular and structural changes may lead to the development of novel therapeutic targets.

## Modifiable vascular signaling pathways

**Figure 3:**
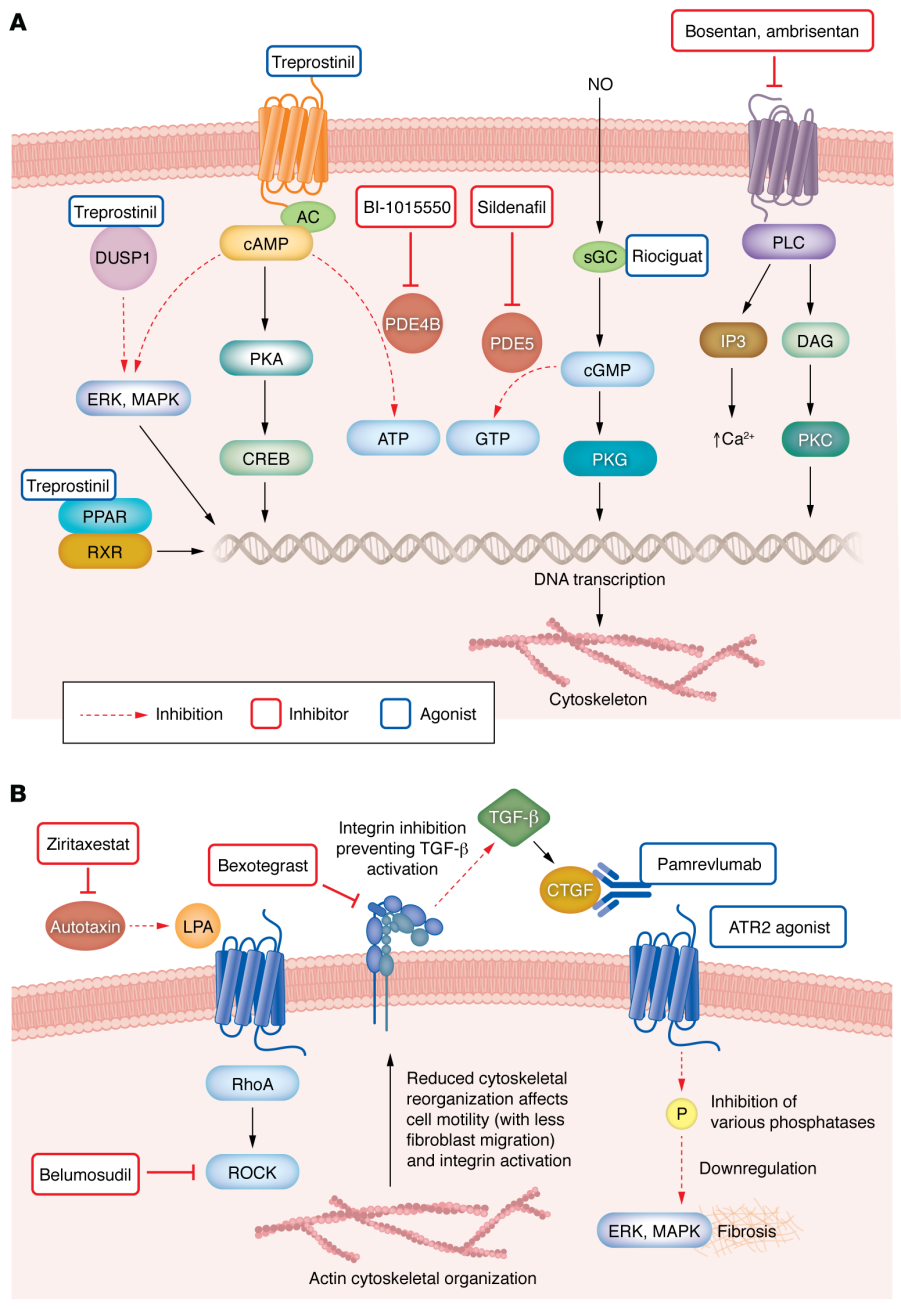
Vascular signaling pathways regulate fibrosis via GPCR, NO, intracellular (PPAR) receptors, and surface integrins in IPF. (**A**) Drugs that may counter fibrosis can act through signaling pathways in a range of vascular cell types, including ECs, VSMCs, and fibroblasts. Fibrogenesis-promoting pathways involve GPCRs or NO and signal through cAMP or cGMP to induce fibrosis-related transcriptional events. Treprostinil (TP) acts on cell surface GPCRs to increase intracellular cAMP, which can affect transcription of actin-encoding genes that affect the cytoskeleton, cell motility, and adhesion. TP can also directly activate intracellular PPAR receptors to modulate gene expression. PDE inhibitors (BI-1015550 and sildenafil) prevent cAMP and cGMP breakdown. cGMP, generated following exposure to endogenous NO, activates PKG, which affects gene transcription, the cytoskeleton, and cell contraction. Stimulators, including riociguat, can also generate cGMP. The ET antagonists bosentan and ambrisentan block GPCRs to reduce intracellular Ca^2+^ concentrations and PKC activity, again modulating gene expression. (**B**) Therapeutics in IPF can signal through pathways affecting TGF-β signaling or other mechanisms promoting profibrotic gene expression. Ziritaxestat blocks autotaxin, from which LPA is generated. LPA induces various profibrotic effects via GPCRs, including increased RhoA activity and actin cytoskeleton rearrangements that promote altered cell motility in a range of cells relevant to fibrosis. Belumosudil preferentially blocks the ROCK2 isoform. The cytoskeleton can activate cell surface integrins, which are implicated in TGF-β activation. Integrins can be directly blocked by bexotegrast. CTGF, which has numerous profibrotic signaling effects, can be neutralized by the monoclonal antibody pamrevlumab. ATR2 agonists affect numerous intracellular phosphatases, which affect downstream profibrotic gene expression.

The key vascular signaling pathways that promote fibrogenesis involve NO or GPCR signaling via cAMP or cGMP to promote transcriptional events (Figure 3A) or inside-out signaling via the α_v_β_1_ integrin (Figure 3B). Several therapeutics targeting various components of these signaling pathways have been assessed with varying degrees of success in IPF trials (Figure 3 and Table 1).

### Endothelial NOS.

NO is a free radical second messenger that is generated by three isoforms of synthase: neuronal, inducible, and endothelial. Endothelial NO synthase (eNOS; also known as NOSIII) is expressed in vascular ECs and produces NO via a calcium/calmodulin pathway. NO subsequently activates soluble guanylyl cyclase (sGC) to generate cGMP, which in turn activates protein kinase G (PKG), leading to modulation/reduction of intracellular calcium concentration, leading to EC permeability, smooth muscle relaxation, and the inhibition of platelet aggregation (60). When eNOS is overexpressed in transgenic mice, the degree of bleomycin-induced subpleural fibrosis is attenuated (61). Furthermore, mice lacking all three NOS isoforms exhibit a worse fibrotic reaction to bleomycin. These fibrotic effects were attenuated in iNOS-null mice that possessed eNOS and in mice supplemented with an NO donor (62), suggesting that NO is protective against fibrosis. However, application of a NO-specific inhibitor attenuated bleomycin-induced fibrogenesis and inhibited angiogenesis by regulation of VEGF and inhibition of PAI-1, indicating that NO serves a complicated role in fibrosis (63). Resolution of bleomycin-induced fibrosis requires eNOS-dependent deactivation of myofibroblasts, and there is evidence of diminished eNOS in aged mice (64). Loss of eNOS may provide one mechanism through which aging promotes IPF.

The sGC stimulator riociguat targets the NO/cGMP pathway and is licensed for use specifically in chronic thromboembolic pulmonary hypertension and also has demonstrable antifibrotic effects in animal models (65, 66). In the RISE-IIP phase II randomized controlled trial (RCT) of 147 patients, riociguat was compared with placebo in patients with PH and interstitial lung disease (ILD), of which IPF was the most common subtype, representing 74% of the treatment arm (67). The trial was terminated early due to earlier mortality and an increased risk of adverse events, including worsening of ILD in the treatment group, and no evidence of any therapeutic benefit.

Inhaled NO gas has recently been shown to improve exercise capacity (as assessed by ability to undertake moderate to vigorous activities) and was well tolerated in patients with ILD and PH (ILD-PH) where IPF represented the largest subgroup of ILD in a phase IIb RCT (68). However, no data on functional markers of fibrosis (e.g., FVC) were measured in follow-up. A randomized trial assessing the effects of NO on dyspnea specifically in IPF patients is under way (ClinicalTrials.gov NCT05052229).

### Endothelin.

Endothelin (ET) is a potent vasoconstrictor with vascular remodeling properties; therefore, ET antagonists are used in the treatment of idiopathic PAH. ETA and ETB receptors are expressed on alveolar epithelial cells and fibroblasts, both of which, under certain disease conditions, are capable themselves of ET synthesis (69). ET can induce fibroblast differentiation, migration, and survival; and epithelial-mesenchymal transition (EMT) and EndMT and ECM production and antagonism of ET can attenuate fibrosis in animal models (70, 71). Similarly, VSMCs when stimulated with interferon in combination with TNF-α acquire the ability to synthesize ET (72–77). ET is increased in plasma and bronchoalveolar lavage (BAL) of patients with IPF and in bleomycin animal models with evidence of increased ET expression in fibrotic tissue particularly in ECs in areas of angiogenesis (78–80).

The dual ETA and ETB receptor antagonist bosentan was compared with placebo in 158 patients with IPF in the BUILD-1 study. Bosentan was well tolerated; however, the trial did not meet its primary endpoint of improvement in 6-minute walk distance (6MWD) (81). However, a trend favoring bosentan was noted toward reduced death and disease progression (although the trial was not appropriately powered to conclude either). A post hoc subgroup analysis suggested that this effect was more pronounced in patients with biopsy-proven IPF. Therefore, the BUILD-3 study was powered to meet these endpoints and enrolled 616 patients with IPF diagnosed by surgical lung biopsy. No significant difference was observed between the bosentan and treatment groups in stabilization of lung function or death (21). Macitentan, another dual receptor antagonist, also did not meet its primary endpoint of change in pulmonary function tests, disease progression, or death in a phase II RCT (22). Assessment of a potent ETA-selective receptor antagonist, ambrisentan, in IPF was terminated early due to worsening disease progression in the treatment (27%) versus control group (17%) (1). These trials demonstrate no beneficial effect of ET receptor antagonists in IPF and suggest that specific targeting of the ETA receptor is harmful. It is, therefore, possible that targeting the ETB receptor may have a beneficial effect in IPF, although specific inhibitors have not yet been developed.

### Cyclic nucleotides and phosphodiesterases.

cAMP is an intracellular messenger formed by adenylate cyclase. In the endothelium, cAMP functions to maintain barrier junction integrity and permeability (through a combined effect with Rho) and vascular smooth muscle tone (82). In human lung fibroblasts, cAMP activation can limit proliferation and ECM differentiation (83). cAMP is degraded by several intracellular phosphodiesterases (PDEs). Dual inhibition of PDE3 and PDE4 can inhibit migration of VSMCs in rats and reverse the vascular remodeling of PH (84), and specific PDE4 inhibition causes less histological fibrosis and collagen accumulation in murine models compared with controls (85, 86).

The nonselective PDE4 inhibitor roflumilast is currently in use in chronic obstructive pulmonary disease and has proven effective in bleomycin mouse models in limiting fibrosis and vascular remodeling (87). However, it is limited by its side effect profile, notably substantial diarrhea. A recent phase II trial of a preferential PDE4B inhibitor, BI-1015550, in patients with IPF proved effective in stabilizing FVC after 12 weeks, and this result was independent of concurrent antifibrotic use (based on median difference in FVC between treatment and placebo groups of 62.4 mL and 88.4 mL in patients with and without concurrent antifibrotic use, respectively) (19). Thirteen percent of patients discontinued therapy in the treatment arm due to diarrhea. The proposed mechanisms of action of the PDE4B inhibitor include an inhibitory effect on fibroblast proliferation and ECM production and an anti-inflammatory component (88). These benefits have made BI-1015550 a promising agent, and it is being further assessed in large phase III studies (NCT05321069).

cGMP is a parallel intracellular second messenger to cAMP with similar functional effects in some systems. cGMP is catabolized by a number of PDE enzymes, with PDE5 being the most therapeutically valuable and a standard target in the treatment of PAH. Inhibition of PDE5 in in vitro models with sildenafil prevents TGF-β–induced EndoMT and VSMC-mesenchymal transition through downstream signaling on ERK1/2 and SMAD, providing further evidence of a beneficial antifibrotic effect of these pathways (89).

In a small open-label study, beneficial effects of sildenafil on 6MWD observed in patients with IPF-PH suggested that enhancing NO/cGMP pathways has therapeutic potential in this condition (90). Subsequently, the STEP-IPF double-blind RCT of sildenafil compared with placebo in 180 patients with advanced IPF (defined by diffusing capacity of carbon monoxide [DLco] of less than 35% of predicted) did not meet its primary endpoint of improvement in 6MWD, although there were improvements in DLco, which was a key secondary endpoint (91). Some subjective secondary endpoints, including quality of life, also showed improvement. The INSTAGE trial, comparing nintedanib as the standard of care combined with sildenafil versus placebo, also did not meet its primary endpoint of change in quality of life as measured by the St. George’s Respiratory Questionnaire (92). However, a reduction of the rate of decline in FVC of at least 5% was observed in the nintedanib plus sildenafil group versus the control arm (31.4% vs. 50.7% of patients; HR 0.56; 95% CI 0.38–0.82). Additionally, a recent cohort study identified a survival benefit in patients with ILD-PH who were treated with sildenafil (93).

### Prostanoids.

Prostanoids comprise a group of lipid mediators including thromboxane, prostaglandin E_2_ (PGE_2_), PGI_2_ (prostacyclin), and PGD_2_, all formed from arachidonic acid (94). Arachidonic acid is liberated from membrane phospholipid in multiple cells by the action of phospholipase A_2_ and converted to PGH_2_ by the action of cyclooxygenase 1 (COX-1) (constitutive) or COX-2 (inducible). PGH_2_ is converted to its respective prostanoid by the action of the site-specific synthase. In the vasculature, prostacyclin is the primary prostanoid produced, largely because of coexpression of COX-1 and prostacyclin synthase by ECs (95). As a counterbalance, platelets release primarily thromboxane since they coexpress COX-1 and thromboxane synthase (96). Prostacyclin is a fundamental antithrombotic mediator and induces vasodilation in some, but not other, vascular beds. Inhibition of COX-2 with NSAIDs, including COX-2–selective medications, is associated with increased risk of cardiovascular mortality due to loss of prostacyclin (97). By contrast, inhibition of COX-1 in platelets (i.e., with low-dose aspirin) is an established preventative therapy for secondary cardiovascular events. Prostacyclin and its analogs have an established role in the therapy of PAH and in the treatment of peripheral vascular disease. Therapeutic formulations that target prostacyclin receptor pathways include iloprost, selexipag, and treprostinil (98).

Prostanoids have long been investigated as possible antifibrotic mediators. Reduced levels of PGE_2_ are found in the BAL fluid from patients with IPF (99). In gene knockout models of fibrosis, mice deficient in the prostacyclin receptor developed worse fibrosis in response to bleomycin as measured by hydroxyproline content and measures of lung mechanics (100). This effect was dependent on COX-2 expression. A prostacyclin receptor–specific agonist applied to human IPF fibroblasts demonstrated an antifibrotic effect, with inhibition of fibroblast proliferation, reduced ECM secretion, and, importantly, a reversal of the myofibroblast phenotype. These effects were mediated by cAMP with proposed downstream mechanisms including hijacking of gene transcription from the TGF-β/SMAD canonical pathway, such as the inhibiting transcription factors YAP and TAZ, which are implicated in transcription of genes including the gene encoding connective tissue growth factor (*CTGF*) (101). cAMP also inhibits the MAPK pathway, which is implicated in fibrosis via a mitogenic effect of PDGF. Upregulation of PKA activity inhibits downstream effectors in this pathway, such as ERK, which is also implicated in fibrogenesis. Inhibition of the ERK pathway can be enhanced when there is a sustained cAMP activity within the cell nucleus as opposed to cAMP activity within the cytosol alone, an effect seen with treprostinil (102). This synthetic prostacyclin analog can also upregulate inhibitors of ERK, notably DUSP1, and inhibit activity of microRNA clusters involved in regulation of this pathway (103). In VSMCs, prostacyclin can inhibit cell proliferation through a cAMP/EPAC/PKA-dependent mechanism and also inhibit vascular smooth muscle cell migration via a cAMP/EPAC/RhoA pathway, which prevents cytoskeletal reorganization (104). In addition, activation of a range of PPAR receptors has an inhibitory effect on TGF-β signaling and fibrogenesis in animal models of fibrosis (105).

PGE_2_ is a central component of the inflammatory response in humans. While a multitude of cells release the wider range of prostanoids, PGE_2_ is considered a critical regulator of inflammation, and inhibition of PGE_2_ at the site of inflammation explains much of the therapeutic benefit of NSAIDs. Animal models and in vitro experiments on human lung fibroblasts have demonstrated reduced production of COX-2–dependent PGE_2_, which may be explained by epigenetic changes in patients with IPF and the milieu of chemokines such as CCL2, which inhibits PGE_2_ release (106). There is also abnormal PGE_2_ receptor (EP1, 2, 3 and 4) expression in fibrotic tissue. PGE_2_ can inhibit fibroblast proliferation and ECM production through the EP2 and EP4 receptors in a cAMP/PKA-dependent manner; however, higher PGE_2_ concentrations can have a profibrotic effect via the EP1 receptor (through downregulation of cAMP) and the EP3 receptor (via increased intracellular calcium). PGE_2_ can also inhibit the effect of TGF-β and the SMAD pathway. Administration of exogenous PGE_2_ in mice has a protective effect against bleomycin-induced fibrosis (107). PGE_2_ deficiency is also important in the increased apoptotic phenotype of lung epithelial cells and apoptosis resistance in fibroblasts in IPF tissue, which contributes to disordered wound healing of the disease (108). There is also evidence that the prostanoid PGD_2_ has a protective effect in bleomycin-induced fibrosis in mice and reduces vascular permeability (109). Stimulation of the PGF2a receptor conversely promotes fibrogenesis (110).

Inhaled treprostinil was evaluated in patients with ILD-PH in INCREASE, a phase III RCT looking primarily at treatment of PH. The trial met its primary endpoint of improvement in 6MWD (16). Interestingly, a post hoc analysis of FVCs measured found that in the 163 patients in the treatment, there was an overall improvement in FVC, and this was most pronounced in the subgroup of patients with IPF (17). A large phase III trial is currently under way investigating treprostinil specifically in IPF (111).

### Rho/ROCK.

RhoA is a GTPase that activates Rho-associated protein kinase (ROCK), leading to phosphorylation of myosin light chains to reorganize the actin cytoskeleton, promoting cell contraction, motility, and adhesion in inflammatory cells, smooth muscle cells (notably VSMCs that are important for regulating vascular tone), and platelets (112, 113). Two isoforms have been identified: ROCK1, expressed ubiquitously, and ROCK2, expressed predominantly in cardiac tissue, pulmonary tissue, and smooth muscle (114). The RhoA/ROCK pathway is implicated in the normal lung wound healing process, facilitating fibroblast and epithelial cell migration in response to receptor signaling by TGF-β, lysophosphatidic acid (LPA), and thrombin/PAR-1, and may also regulate profibrotic gene expression (115, 116). The activity of RhoA/ROCK is enhanced in IPF tissue (117). In the endothelium, activation of RhoA/ROCK signaling by LPA is responsible for generation of vascular leak by generating cellular contraction and disrupting cell-cell and cell-matrix adhesion (118). Extravascular leak of profibrotic mediators, including thrombin (itself a ROCK activator in epithelium and fibroblasts), propagates the fibrotic process, thus driving fibrogenesis. In pulmonary ECs in response to hypoxia, ROCK may also downregulate expression of eNOS, which, as described, is involved in the generation of both fibrosis and PH (119).

A mechanistic link has been demonstrated in shared pathways in RhoA activation and PDE4 via A-kinase anchoring protein 13 (AKAP13) (120). AKAP13 activates PDE4, reducing protein kinase activity in addition to having a RhoGEF function whereby it can phosphorylate and activate RhoA, suggesting that AKAP13 may be a master regulator of fibrotic responses.

In animal models of fibrosis, inhibition of ROCK using fasudil (which has clinical applications in the management of subarachnoid hemorrhage due to its vasorelaxant properties) and the experimental compound Y-27632 attenuates fibrosis and vascular remodeling (112, 121). In gene-deleted animal models, bleomycin-induced fibrosis is attenuated when either ROCK isoform is deleted, indicating that both enzymes are implicated in fibrogenesis (122). This is relevant as selective ROCK inhibition may be sufficient to inhibit fibrosis and avoid complications such as hypotension. Selective inhibition of ROCK2 with Slx-2119 downregulated profibrotic gene expression in a range of fibrotic effector cells including VSMCs in in vitro models (123). The selective ROCK2 inhibitor belumosudil has been evaluated in a phase I trial of patients with IPF, where it was well tolerated and slowed decline in lung function (124). Belumosudil is currently the subject of a phase II trial (NCT02688647); although initial results have not been published, the results posted on ClinicalTrials.gov suggest that FVC remains unchanged.

### Coagulation cascade.

There are a number of potential mechanisms through which fibrosis and abnormal clotting may occur. Tissue factor initiates the extrinsic coagulation pathway, is highly expressed by alveolar epithelial cells in patients with IPF, and generates lung-tissue fibrin deposits, which serve as a platform for inflammatory cells and profibrotic cytokines, enhancing their accumulation at sites of injury (125). This environment favors an imbalance in the system toward the pro-coagulation pathway. Activation of the coagulation cascade generates multiple proteases and thrombin, which has profibrotic actions in part due to its action on PAR receptors, particularly PAR-1. PAR receptors are present on a range of cells including ECs and fibroblasts, affecting EC barrier integrity and promoting release of PDGF and CTGF, and promoting fibroblast differentiation (126).

Similarly, elevated levels of both factor VIII, a marker of EC injury that is implicated in thrombosis, and fibrin degradation products, including D-dimers, are seen in patients with IPF, suggesting exuberant coagulation (127). Factor X expression is also increased in fibrotic human lung tissue and in mouse models of fibrosis (128). This protein is implicated in myofibroblast differentiation via activation of the PAR-1 receptor, which is highly expressed in fibroblastic foci. Activation of PAR-1 also increases RhoA activity, which can activate TGF-β from its latent complex via α_v_β_6_ integrin (115).

Despite the evidence of disrupted clotting in IPF, the STEP-IPF study, comparing warfarin (an inhibitor of factors II, VII, IX, and X) with placebo, demonstrated harm, and this detriment was due to accelerated fibrosis rather than bleeding complications (129). Recent registry data support this finding, with warfarin being associated with reduced transplant-free survival; however, treatment with newer direct oral anticoagulants, which are direct factor Xa inhibitors, was not associated with a reduction in survival (130). These results suggest that selective inhibition of factor Xa may have antifibrotic potential. To provide further evidence for the benefits of targeting specific coagulation pathways, the profibrotic effects of thrombin acting via the PAR-1 receptor mediated through α_v_β_6_ and TGF-β can be inhibited using the direct thrombin inhibitor dabigatran in a murine model (131).

### Other pathways.

Other EC-relevant pathways that have been investigated in PF include (a) autotaxin, the enzyme responsible for generation of the profibrotic lipid mediator LPA, which also has phosphodiesterase activity and is highly expressed by ECs; (b) the integrins, including α_v_β_1_ and α_v_β_6_, which are implicated in PF and have important effects on EC function (132); (d) CTGF, which is potently fibrogenic and contributes to the development of IPF-PH; and, finally, (e) the renin-angiotensin system — in particular the ATR2 receptor — agonism of which attenuates vascular remodeling in models of PH (133). Specific targeting of these pathways has been or is being trialed (Table 1 and Figure 3B).

## Disease associations

It is evident from cell and molecular biology that the vasculature, and related signaling pathways, have an important role in the development of IPF. If the vasculature is playing a prominent role in the pathogenesis of IPF, one would hypothesize that there would be substantial systemic disease associated with IPF. While PH is a well-recognized complication of IPF, other comorbidities are gaining prominence. Indeed, systemic hypertension and diabetes are common comorbidities in IPF (134), while coronary artery disease and thromboembolic disease are common causes of death (135, 136) (Table 2).

## Conclusions

The epithelium — the primary site of initial insult in IPF — sits in close proximity to the endothelial layer in the alveolus, separated only by a thin basement membrane, which itself is abnormal in the condition. Injury to the former will undeniably affect the latter. There is compelling evidence from various in vivo, in vitro, and ex vivo models that aberrant endothelial responses occur with resultant loss of integrity of the basement membrane, vascular remodeling, and the generation of vascular signals further driving ECM deposition, fibrosis, and parenchymal lung damage. Whether it is the epithelial signal to the endothelium that initiates this process in its entirety, or whether the endothelium is the primary culprit, remains an area for development in the field of fibrosis research. However, given the findings, one cannot ignore the potential role of the circulation in the pathobiology of IPF.

The numerous disrupted vascular-relevant signaling pathways present in IPF are now being explored in greater detail and mapped more clearly. The specific targeting of these pathways — especially with prostanoid agents and phosphodiesterase inhibitors — represents an expanding chapter in the treatment of this challenging disease. As closer associations between IPF and numerous more common cardiovascular and metabolic conditions are made, a deeper understanding of the disease and how to treat it is likely to ensue. Therefore, strategies that target endothelial repair by focusing on reprogramming abnormal metabolic responses may ultimately provide an opportunity to prevent the progression of, or potentially even reverse, the fibrotic response.

## Figures and Tables

**Table 1:**
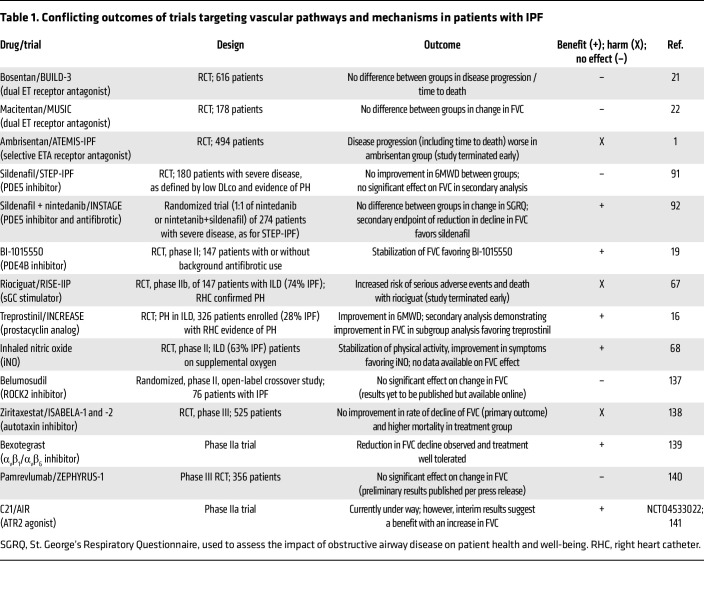
Conflicting outcomes of trials targeting vascular pathways and mechanisms in patients with IPF

**Table 2:**
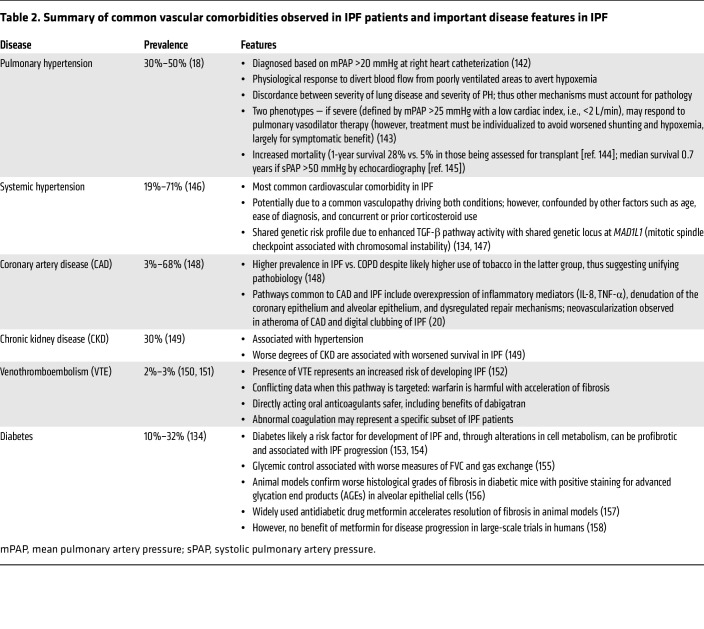
Summary of common vascular comorbidities observed in IPF patients and important disease features in IPF

## Version 1 09/15/2023

Electronic publication
